# Neural activation following offensive aggression in Japanese quail

**DOI:** 10.1242/bio.038026

**Published:** 2018-11-07

**Authors:** Cornelia Voigt, Katharina Hirschenhauser, Stefan Leitner

**Affiliations:** Max Planck Institute for Ornithology, Department of Behavioural Neurobiology, D-82319 Seewiesen, Germany

**Keywords:** Immediate early genes, Japanese quail, Aggression, Pallium

## Abstract

Aggression is a fundamental part of animal social behaviour. In avian species, little is known about its neural representation. In particular, neural activity following offensive aggression has not been studied in detail. Here, we investigated the patterns of brain activation using immediate-early gene (IEG) expression in male Japanese quail that showed pronounced aggressive behaviours during a 30 min male–male interaction and compared them to those of males that did not interact with a conspecific. In aggressive males, we found a massive induction of the IEG *ZENK* in pallial brain structures such as the intermediate medial mesopallium, the caudomedial mesopallium and the intermediate medial nidopallium. To a lesser extent, activation was observed in subpallial areas such as the nucleus taeniae of the amygdala and in the medial portion of the bed nucleus of the stria terminalis. Our data suggest that the modulation of aggressive behaviour involves the integration of multisensory information.

## Abstract (precis)

**Summary:** The manuscript describes the pronounced activation of the immediate early gene *ZENK* in the pallium of male Japanese quails following offensive aggressive behaviour.

## INTRODUCTION

Agonistic behaviour constitutes a key component of vertebrate societies and it comprises all instances of attack, threat, defence, escape and submission (Szekely et al., 2010). First attempts to localise brain sites responsible for agonistic behaviour patterns were made by studies employing electrical stimulation (Akerman, 1966; Maley, 1969; Phillips and Youngren, 1971). For example, in freely moving Mallard ducks (*Anas platyrhynchos*), aggressive reactions, i.e. directed attacks, could be elicited by stimulating the arcopallium and several areas of the hypothalamus, whereas escape reactions could primarily be elicited from diencephalic brain regions and the midbrain (Maley, 1969). Subsequent studies, employing a wide variety of techniques, have identified neural sites, which are activated not only by aggressive but also by other social behaviours such as mating behaviour or parental behaviour. These findings led to the view that sexual, aggressive and other types of social behaviours are controlled by a common neural circuit of limbic brain structures called the social behaviour network (Newman, 1999). Based on evidence from hodological, neurochemical and functional studies, this concept, originally proposed for mammals, was later extended to reptiles (Crews, 2003), birds and fish (Goodson, 2005; but see Goodson and Kingsbury, 2013).

Recent studies in birds, measuring the activity of immediate-early genes (IEGs) such as *c-Fos* and *ZENK*, have identified subpallial and hypothalamic brain areas such as the lateral septum (LS) and the medial preoptic area (POM) and the paraventricular nucleus (PVN), all belonging to the social behaviour network, which show increased neural activity following male–male interactions or simulated territorial intrusions (Goodson and Evans, 2004; Goodson et al., 2005; Xie et al., 2010). These studies, however, did not differentiate between males expressing mainly aggressive or defensive behaviours. Consequently, the pattern of neural activation in these individuals likely resulted from showing aspects of both (Xie et al., 2010). The aim of the present study was to investigate which brain areas are activated when males engage in offensive aggression. Therefore, we analysed the gene expression level of the IEG *ZENK* in the brain of male Japanese quail (*Coturnix japonica*) that showed a significantly higher frequency of aggressive behaviours than their opponents during a 30 min male–male interaction in comparison to control males that did not interact with a conspecific. Japanese quail form dominance relationships among the males of a group and respond vigorously to the presence of unfamiliar males, which makes this species well suited for studying the mechanisms of agonistic behaviours (Mills et al., 1997).

## RESULTS

### Quantification of aggressive behaviour during the 30 min male–male interaction

Males began to attack on average within 2 s (median, range 1–70 s) and in all dyads, the male that initiated the interaction was the more aggressive individual. Within the pairs of opponents, males differed significantly in the frequencies of chases (Wilcoxon test, W=0, *N*=7, *P*=0.018) and attacks (Wilcoxon test, W=0, *N*=7, *P*=0.018) and the frequency of crows was also higher in the more aggressive counterpart but the difference failed to be significant (Wilcoxon test, W=0, *N*=7, *P*=0.06). In two out of seven pairs, both males displayed chases and attacks towards each other. Generally, the inferior males squatted or attempted to escape. The significantly more aggressive males from each dyad were subjected to further analysis and in the following they are termed as ‘aggressive’ males as opposed to control males, which experienced no interaction with a male conspecific prior to euthanasia.

### Morphological differences between aggressive and control males

Control males were significantly heavier than aggressive males (control: 286.3±4.7 g; aggressive: 257.4±7.5 g; *t*=3.27, *P*=0.007) but the area of the cloacal gland, an androgen-sensitive structure, was smaller than in aggressive males (control: 300.4±10.0 mm^2^; aggressive: 358.5±16.0 mm^2^; *t*=3.09, *P*=0.009). No group differences were found regarding testes mass (*t*=0.36, *P*=0.723) and brain mass (*t*=0.77, *P*=0.46).

### Differential *ZENK* expression between aggressive and control males

**Fig. 1.**
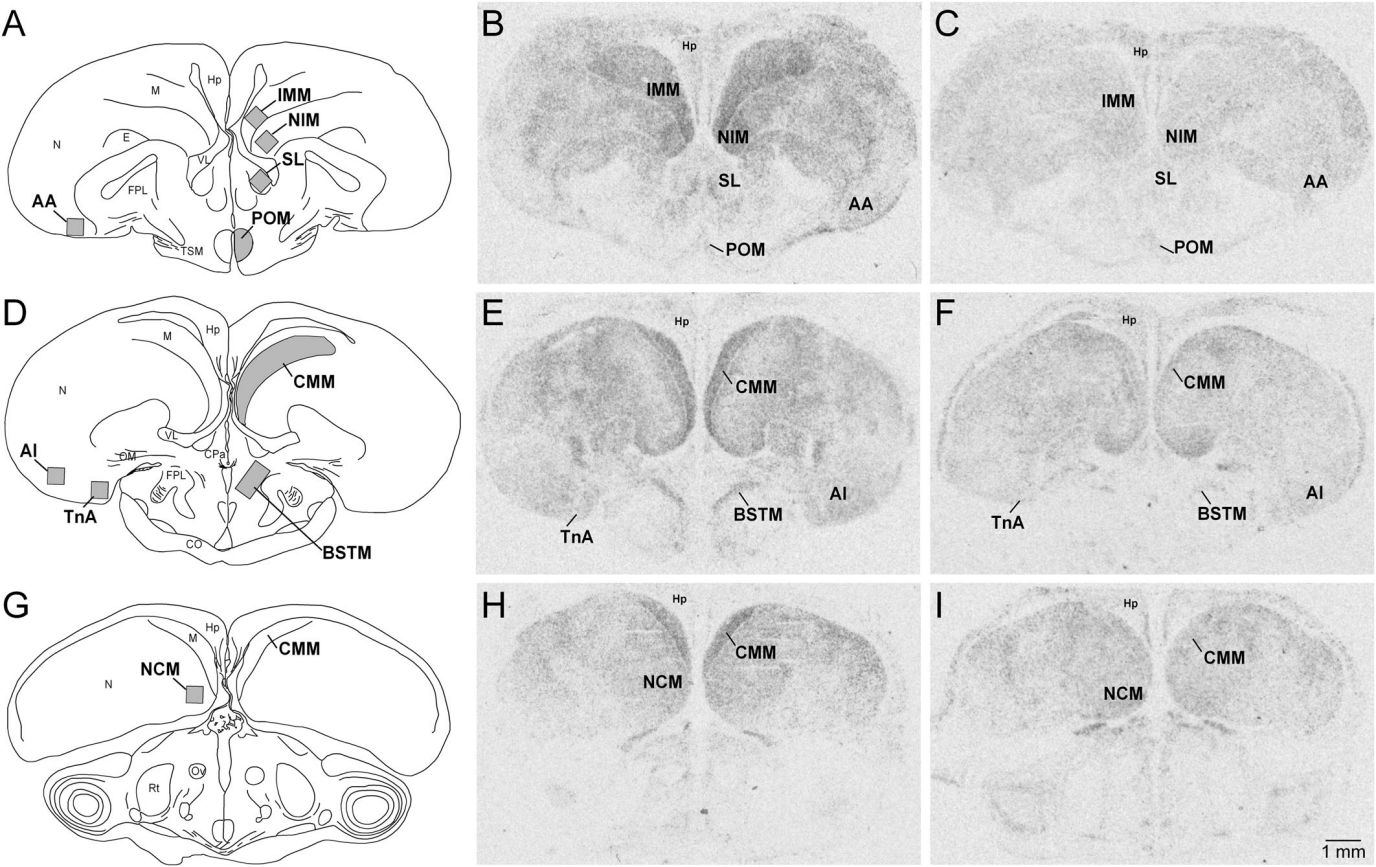
**Autoradiograms of coronal sections through the quail brain illustrating the expression of *ZENK* mRNA visualised by *in situ* hybridisation.** (A,D,G) Schematic drawings showing the areas where *ZENK* expression was quantified. Sections are in rostral to caudal order. (B,E,H) *ZENK* expression in an aggressive male. (C,F,I) *ZENK* expression in a control male. Abbreviations: AA, arcopallium anterius; AI, arcopallium intermedium; BSTM, bed nucleus of the stria terminalis; CMM, mesopallium caudomediale; CO, chiasma opticum; E, entopallium; FPL, fasciculus prosencephali lateralis; Hp, hippocampus; IMM, mesopallium intermediomediale; M, mesopallium; N, nidopallium; NCM, nidopallium caudomediale; NIM, nidopallium intermedium, pars medialis; OM, tractus occipito-mesencephalicus; Ov, nucleus ovoidalis; POM, nucleus preopticus medialis; Rt, nucleus rotundus; SL, lateral septal nucleus; TnA, nucleus taeniae of the amygdala; TSM, tractus septopallio-mesencephalicus; VL, ventriculus lateralis.

**Fig. 2.**
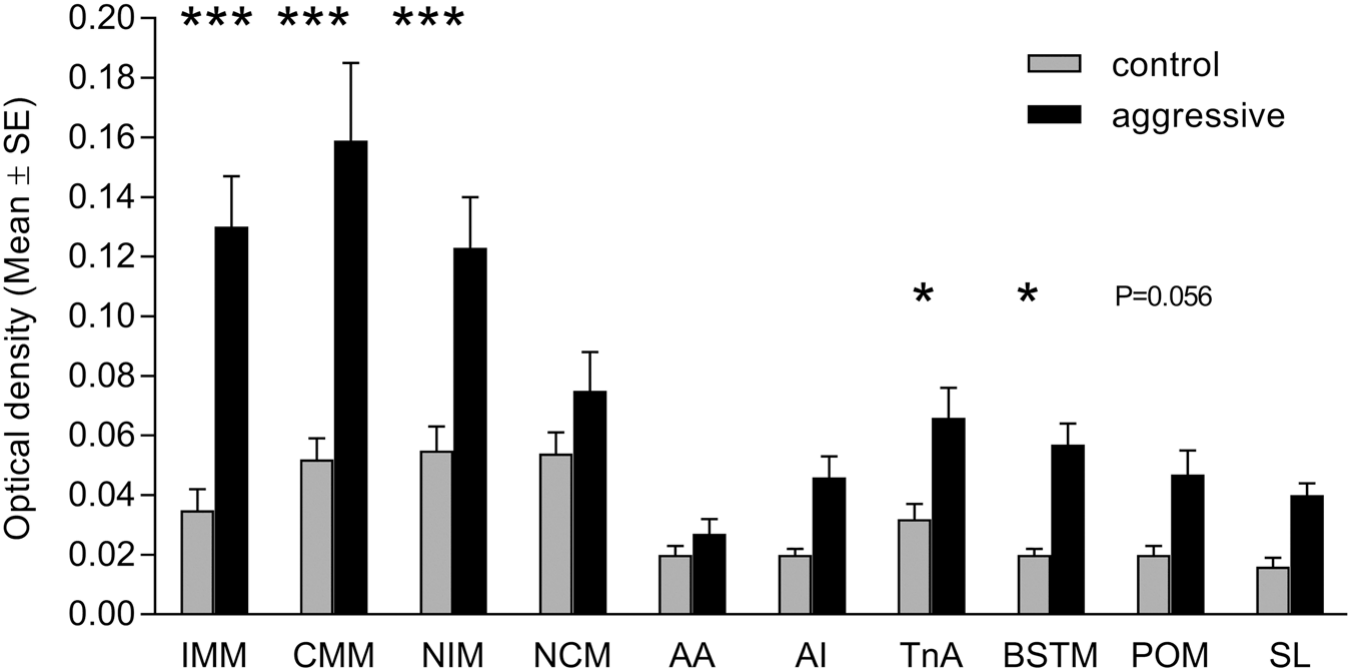
**Average optical density (mean±s.e.) of the *ZENK* hybridisation signal for different forebrain regions of** **male Japanese quails.** Aggressive males (*N*=7) and control males (*N*=7) (***P<0.001).

We quantified the gene expression levels of *ZENK* in pallial, subpallial and hypothalamic regions (Fig. 1). Analysis of the average optical density in the ten different cell groups revealed significant group differences (treatment: *F*_1,12_=18.15, *P*=0.0011; brain region: *F*_9,108_=35.03, *P*<0.0001; interaction: *F*_9,108_=11.82, *P*<0.0001). *Post hoc* tests showed that most pronounced differences in the intensity of the hybridisation signal between both groups of males were found in the mesopallium and nidopallium compared to other brain regions. Aggressive males showed a massive *ZENK* induction in the intermediate medial mesopallium (IMM), in the caudomedial mesopallium (CMM) and in the intermediate medial nidopallium (NIM). *ZENK* expression was also significantly higher in the bed nucleus of the stria terminalis (BSTM) and the nucleus taeniae of the amygdala (TnA) of aggressive males compared to controls (Figs 1B,E,H and 2). No signal was detected in the Field L and the entopallium.

## DISCUSSION

The major finding of our study is the pronounced activation of pallial brain areas following the performance of offensive aggressive behaviours. Induction of IEG gene and/or protein expression in the avian pallium has been previously detected in relation to a variety of different stimuli such as auditory perception (Mello and Clayton, 1994; Leitner et al., 2005; Terpstra et al., 2005), filial imprinting (Long et al., 2002; Thode et al., 2005), passive avoidance learning (Anokhin et al., 1991) and male sexual behaviour (Meddle et al., 1997; Tlemçani et al., 2000). The present study is the first to report IEG expression in specific pallial brain regions in response to offensive aggression. By analysing *ZENK* expression in males that emerged as significantly more aggressive than their opponents after a 30 min male–male interaction we ensured that the brain activity reflects mainly aggressive rather than defensive behaviour.

We cannot rule out completely that the pronounced neural activation occurred in response to other stimuli than engaging in aggressive behaviour, such as locomotion, but in the light of previous findings in the same species it seems rather unlikely. The expression of the IEG *c-Fos* in hypothalamic areas or in the mesopallium does not significantly increase if males are kept in their home cage, if they are placed into an empty testing arena or if they are placed into a testing arena with a female but no copulatory behaviour is expressed. Also, a high frequency of crowing does not induce *c-Fos* expression in these brain areas (Meddle et al., 1997; Charlier et al., 2005; Taziaux et al., 2006). It was also shown in chickens (*Gallus gallus*) that locomotor activity alone does not activate pallial or hypothalamic regions (Xie et al., 2010).

Our study shows that males that acted aggressively towards male conspecifics showed massive activation of brain regions within the mesopallium and nidopallium. The mesopallial areas most upregulated are the auditory subdivision comprising the caudal mesopallium and the multisensory subdivision comprising the medial part of the intermediate ventral mesopallium. Tract-tracing studies in pigeons (*Columba livia*) have shown that the latter receives somatosensory, auditory and visual input from thalamic nuclei and pallial regions and connects with the arcopallium, nidopallium and lateral striatum. The intermediate medial nidopallium receives the same types of sensory input as the intermediate medial mesopallium and both regions are strongly interconnected (Atoji and Wild, 2012). Auditory stimulation during filial imprinting in guinea fowl chicks and Japanese quail chicks leads to simultaneous activation of both areas (Maier and Scheich, 1983; Kabai et al., 1992). However, the sensitive period for imprinting ends about one week after hatching. Our data show that this neural circuit is still maintained in adulthood although in a different context. For example, in adult crows (*Corvus brachyrhynchos*) the medial mesopallium and nidopallium were activated during discrimination of visual stimuli such as human faces, which the birds had learnt to associate with a threatening experience (Marzluff et al., 2012). In contrast, the pattern of neural activation differed when the crows were exposed to a stimulus which represented an innate fear such as the sight of a predator (Cross et al., 2013). This confirms that these higher order sensory areas are activated when the situation involves learning new information or the retrieval of learned information. Therefore, in quail that were engaged in a male–male interaction, not only the confrontation with a new opponent but, additionally, the memory of previous visual, acoustical or physical encounters with a male conspecific could have been responsible for the activation of the pallial regions. It is also possible that the exposure to a threatening situation, i.e. exposure to a male conspecific, induced a state of arousal (Bischof and Herrmann, 1988).

In the present study, performance of aggressive behaviour also activated brain areas considered to be part of the vertebrate social behaviour network (Goodson, 2005), although to a much lesser extent. The subpallial TnA and BSTM, parts of the avian extended medial amygdala and homologous to the extended medial amygdala of mammals, showed significant *ZENK* induction in aggressive males (and not in control males). Activation of these regions in the context of offensive aggression is well documented in rodents (Kollack-Walker and Newman, 1995; Delville et al., 2000; Davis and Marler, 2004). In birds, however, the evidence is inconsistent. While activation of BSTM was found in male song sparrows (*Melospiza melodia*) engaging in territorial defence (Goodson et al., 2005), a 20 min male–male interaction in chickens did not elicit an increase in IEG expression in BSTM and TnA (Xie et al., 2010). Neural correlates of aggression were found in both BSTM and TnA in large-billed crows (*C. macrorhynchos*; Nishizawa et al., 2011). In quail, the visual presentation of another male conspecific resulted in activation of Fos immunoreactivity in SL but not in other regions (Taziaux et al., 2006). However, TnA and BSTM are activated during male copulatory behaviour in this species (Charlier et al., 2005; Taziaux et al., 2006), and together with the present findings it confirms that these brain areas are part of circuits mediating different types of social behaviour.

In conclusion, our data demonstrate that agonistic behaviour activates a neural network of pallial and subpallial brain regions. Further studies are needed to discriminate between the specific social stimuli leading to region-specific neural activation, such as those requiring memory or learning.

## MATERIALS AND METHODS

### Animals

This study was carried out in August 2010 with sexually mature, gonadally intact male Japanese quail that were purchased from a local breeder in Austria. Birds were housed in separate cages (110×110×100 cm) under a long-day photoperiod (16 h light:8 h dark). Food and water were available *ad libitum*. All males were in acoustical contact with each other and had no physical interaction with other males for at least 10 weeks. Birds were randomly assigned to one of two treatments. Control males (*N*=7) were kept singly in their home cages throughout the study. Experimental males (*N*=14) were subjected to a single 30 min male–male interaction. These males had not physically interacted with each other previously. For neuroanatomical analysis of *ZENK* expression, the males that were significantly more aggressive than their opponents (*N*=7) and the control males (*N*=7) were used. Before being euthanised, the area of the cloacal gland, an androgen-sensitive structure (Sachs, 1967), was measured with a calliper to the nearest millimetre (area=largest length×largest width). The body mass was recorded to the nearest gram. At the time of brain collection, the mass of the testes and of the brain was recorded to the nearest milligram.

### Experimental protocol of male–male interaction

Males were allowed to interact in dyads in a neutral area (110×110×100 cm) for a period of 30 min. Pairs of opponents were assigned randomly. Behaviour was sampled by two observers and simultaneously recorded using a digital Handycam DCR-TRV8E (Sony Electronic Industries) mounted on a tripod. We recorded the latency to first attack, which opponent had initiated the fight and the frequencies of chases, attacks (head, neck and body grabs) and crows. Chases: bursts of locomotion of the subject towards the fleeing opponent. Attacks: the subject grabs or pecks at the opponent's neck, head or body. Crows: the subject stands upright and produces the species-typical vocalisation. Based on quantification of these behaviours, from each pair of opponents the more aggressive male was identified. Immediately after termination of the interaction, the latter was euthanised and the opponent was returned to its home cage. Control males were handled for 5 min by the experimenter, returned to their home cage and euthanised 30 min later.

### Neuroanatomical analysis

Birds were euthanised by decapitation, their brains were removed and stored at −80°C. Frozen brains were cut on a cryostat into 30 µm coronal sections (from the level of the tractus septopallio-mesencephalicus to the third nerve). The plane of the sections was adjusted to match as closely as possible the plane of the quail brain atlas (Baylé et al., 1974). Sections were mounted onto Superfrost Plus slides (Menzel-Gläser, Braunschweig, Germany) and stored at −80°C until analysis.

### Cloning of the quail *ZENK* cDNA probe

The cloning and characterisation of a region of the quail *ZENK* gene (1.1 kb) has been described previously (Long and Salbaum, 1998). Based on this sequence information we cloned an 840 bp fragment, which is identical to nucleotides 127–967 of the quail sequence (Genbank no. AF026083).

### *In situ* hybridisation

The expression of *ZENK* in brain sections was detected with antisense RNA probes labelled with 35S-CTP. Labelling of the probes with 35S-CTP (1250 Ci/mmol; Perkin Elmer, Rodgau, Germany) was performed using the Riboprobe System (Promega). Our *in situ* hybridisation procedure followed a previously published protocol (Whitfield et al., 1990) with modifications as previously described in detail (Gahr and Metzdorf, 1997). For signal detection, sections were exposed to autoradiographic film (Kodak Biomax MR, Rochester, USA). Brains from both groups of males were run through the entire procedure at the same time and sections from both were placed on each autoradiographic film to avoid any possible effect of small differences in procedures on the observed differences in expression level. No labelling was observed with the sense probe.

### Data analysis

Images from autoradiograms were scanned with an Epson Perfection V750 Pro scanner and analysed using the image analysis software ImageJ 1.50i (NIH, USA; http://rsb.info.nih.gov/ij/). The system was calibrated by using a calibrated optical density step tablet (T2115CC; Stouffer Industries, Inc., Mishawaka, USA) and a calibration curve was fitted with the Rodbard function of ImageJ [y=d+(a-d)/(1+(x/c)^b)]. This calibration was applied to all images and extended beyond the darkest spot to be measured in the autoradiograms so that the signals that were measured did never reach saturation. The following regions were quantified: anterior arcopallium (AA), intermediate arcopallium (AI), intermediate medial mesopallium (IMM), caudal medial mesopallium (CMM), intermediate medial nidopallium (NIM), caudomedial nidopallium (NCM), bed nucleus of the stria terminalis (BSTM), medial preoptic nucleus (POM), lateral septum (SL) and the nucleus taeniae of the amygdala (TnA). Brain regions were identified using the atlas of the quail and the chicken (Baylé et al., 1974; Kuenzel and Masson, 1988). The *ZENK* expression level in regions of interest was measured in three subsequent sections, respectively, either by delineating the entire area with the computer mouse on the screen (CMM, BSTM, POM) or within a standard frame of 0.5×0.5 mm for SL and of 0.7×0.7 mm for all other regions. The average optical density (OD) was calculated by the built-in function of the software. Background optical density of the film was measured in a rectangular area (1 mm^2^) in the same image immediately outside of the brain section of interest. Final OD measurements were obtained by subtracting the film background OD value from the OD value of the region of interest.

### Statistical analysis

We employed a REML-model using JMP software (SAS Institute GmbH, Böblingen, Germany) with treatment and brain region as fixed factors and bird ID as random factor. *Post hoc* analyses were performed using the ‘test slices’ comparison in JMP. Morphological data were analysed using unpaired *t*-tests. Differences in agonistic behaviours between males during the 30 min male–male interaction were tested using the Wilcoxon test for matched pairs. Differences with *P*-values <0.05 were considered as significant.

### Ethics

All experiments were carried out in accordance with the EU Directive 2010/63/EU for the use of laboratory animals.
